# NIH-supported implementation science and nutrition research: a portfolio review of the past decade

**DOI:** 10.3389/fpubh.2023.1235164

**Published:** 2023-10-17

**Authors:** Susan Vorkoper, Ariella R. Korn, Padma Maruvada, Holly L. Nicastro, Scarlet Shi

**Affiliations:** ^1^Fogarty International Center, National Institutes of Health, Bethesda, MD, United States; ^2^Cancer Prevention Fellowship Program, Implementation Science Team, Division of Cancer Control and Population Sciences, National Cancer Institute, National Institutes of Health, Bethesda, MD, United States; ^3^Division of Digestive Diseases and Nutrition, National Institute of Diabetes and Digestive and Kidney Diseases, National Institutes of Health, Bethesda, MD, United States; ^4^Office of Nutrition Research, National Institutes of Health, Bethesda, MD, United States; ^5^Division of Cardiovascular Sciences, National Heart, Lung, and Blood Institute, National Institutes of Health, Bethesda, MD, United States

**Keywords:** nutrition, implementation science, research, grants, NIH

## Abstract

**Objective:**

This portfolio analysis aims to describe the scope of NIH-funded extramural research grants at the intersection of nutrition research and implementation science (IS) from 2011 to 2022 and to offer insights into future research opportunities relevant to the Strategic Plan for NIH Nutrition Research 2020–2030.

**Methods:**

A portfolio analysis of funded grants using NIH reporting systems was conducted to identify nutrition research and IS awarded between fiscal years 2011 and 2022. The authors screened the titles and abstracts for inclusion criteria: research and career development awards involved a nutrition and/or dietary intervention and measured a stated implementation outcome or used an IS theory, model, or framework.

**Results:**

In total, 33 NIH-funded awards met the inclusion criteria. Almost half of the awards (48.5%) were investigator-initiated research projects compared to research career awards and cooperative agreements. While studies were predominantly conducted in the United States, 15.2% were conducted in low- and middle-income countries in Africa, Latin America, and Asia. Adults aged 19–64 years and children aged 2–11 years represented most of the study populations (45.5 and 15.2%, respectively). Studies provided nutrition/dietary guidelines and created culturally tailored interventions, which were then adapted in collaboration with community partners in schools, hospitals, and religious settings. The most cited IS outcomes were feasibility, costs, adoption, and acceptability. Sixteen awards (48.5%) used an IS theory, model, or framework to guide their work.

**Discussion:**

The findings show the breadth of NIH-funded nutrition and implementation research and highlight potential research opportunities.

## Introduction

For nutrition and dietary research, implementation science (IS) holds promise for moving the investments in evidence-based interventions into practice (e.g., changing dietary behaviors and environments) (1, 2), sustaining adherence to those changes over time, and implementing strategies to scale and disseminate these interventions (1–3). IS addresses barriers to effective implementation, tests innovative approaches to advancing health programming, and develops and tests implementation strategies to improve diet and nutrition interventions' reach, uptake, and scale-up, among other outcomes (4–6).

The intersection of biological, behavioral, psychosocial, sociocultural, and environmental factors may act additively or cumulatively to improve or impede individuals' chances to change their dietary behaviors and sustain those changes over time (7). Thus, interventions need to target many aspects of the food environment (e.g., food security, food systems, and advertising) while accounting for participants in the context of their environments to gain insights that could improve long-term healthy behaviors. By using multilevel or systems approaches and incorporating metrics related to implementation, IS not only focuses on traditional outcomes such as efficacy and effectiveness but also acknowledges the fundamental role the context plays in nutrition and dietary research (8). Therefore, using IS designs and methodologies is essential for understanding and promoting effective and ready-to-adopt nutrition interventions.

The U.S. National Institutes of Health (NIH) 2020–2030 Strategic Plan for Nutrition Research recognizes the innovative role that IS can play in advancing nutrition research and sets the priorities in the plan (9). A recent commentary by members of the NIH Implementation of Nutrition-related Programs, Practices, and Behaviors working group highlights opportunities to stimulate IS in nutrition research in alignment with this strategic plan across three areas: (a) advancing consideration of implementation and dissemination early in the design of interventions to facilitate opportunities for equitable scale-up and sustainability of evidence-based interventions, (b) developing and testing strategies for equitable implementation of nutrition and diet interventions in health care and community settings, and (c) building and strengthening the infrastructure, capacity, and expertise needed to increase use of IS in clinical and community nutrition research to swiftly move the research into practice (10).

To build on this effort, the NIH Implementation of Nutrition-related Programs, Practices, and Behaviors working group conducted a portfolio analysis of research on the implementation of nutrition interventions funded through the NIH Institutes, Centers, and Offices (ICO). Portfolio analyses of funded research are important tools to provide insights into current interests and potential needs, challenges, and future trends in a given field. This portfolio analysis descriptively examines the funded grant mechanisms for nutrition and IS grants, the characteristics of the diet and nutrition interventions and study populations, and the aspects of implementation research, including implementation outcomes and the use of established IS frameworks. We use the NIH definition of IS, which is the study of scientific methods that facilitate evidence-based research findings into practice and is inclusive of dissemination research (11). The goal of the analysis is to describe the extent, range, and nature of the funded NIH research in nutrition and IS in the past decade, identify research gaps, and consider how to support the opportunities outlined in the commentary (10).

## Methods

We conducted a search to identify nutrition research that used an implementation research approach. We searched an NIH internal reporting system, Query View Report (QVR), using the NIH's Research, Condition, and Disease Categorization (RCDC) system definition of nutrition research. RCDC uses sophisticated text data mining algorithms in conjunction with NIH-wide definitions to create a “fingerprint” that matches projects to research spending categories. Within the nutrition research category, we further conducted a search using keywords to identify awards funded in Fiscal Years 2011–2022 (from October 1, 2010 to September 30, 2022). Search terms included implementation research, implementation science, implementation strategy, implementation trial, annual implementation plans, clinical implementation, dissemination of results, dissemination research, dissemination trial, program dissemination, prevention dissemination research, research data dissemination, and research dissemination. Only the funded awards were exported and downloaded.

We included research projects awarded by the NIH since 2010 a Fellowship Grant (Fs), a Career Development Award (Ks), a Research Project or Center Grant (P01s), a Research Grant (Rs), or a Cooperative Agreement (US). These funding mechanisms constitute most NIH-supported grants, including career development, exploratory, hypothesis testing/generating, and resource-generating projects. Training grants (e.g., D43, D71, and T32) were excluded. Further, the projects had to address some aspects of a nutrition and/or dietary intervention and include consideration and/or measurement of at least one stated implementation outcome (i.e., acceptability, adaptation, adoption, appropriateness, costs, feasibility, fidelity, penetration, reach, sustainability, and scale-up) (12–14) or use an established IS theory, model, or framework (Appendix A, Codebook). The selected outcomes expand on Proctor's original Implementation Outcome Framework from 2011 to integrate (12, 14) those identified by Reilly in 2020 (13) related to the RE-AIM (Reach, Effectiveness, Adoption, Implementation, Maintenance) Framework.

Seven reviewers (AB, ARK, PM, HLN, AO, SS, and SV) screened the publicly available titles and abstracts of the identified awards with the above inclusion criteria using a dual-independent approach (two reviewers per award). Disagreements in screening between two reviewers were resolved by a third reviewer. Six reviewers (ARK, PM, HLN, AO, SS, and SV) then extracted data from those that were included, again using a dual-independent approach. The data were extracted into Microsoft Excel using an established codebook (Supplementary material A). We collected the following information from each award: demographics (e.g., age) of the population studied; location, including country and different settings; nutrition and dietary-related behaviors; implementation framework or theory used (if applicable); IS research focus areas or phases (i.e., IS measurement development; pre-implementation; implementation process description; implementation strategy testing; dissemination strategy testing; de-implementation; sustainability; and scale-up) (15); implementation outcome(s); study design; implementation strategy; type of intervention; and health disparities addressed. Disparities were included if the research focus has any of the following: minority health that included racial and/or ethnic groups who are usually underrepresented in biomedical research; health disparities that addressed health differences that adversely affect disadvantaged populations, based on factors such as higher disease burden, risk factors, condition-specific symptoms, and/or other categories of health outcomes; or health equity, an intervention on a social determinant of health and/or to take an equity approach and/or include a health equity outcome and that addressed an NIH disparity population (i.e., racial/ethnic minority, underserved rural populations, socioeconomically disadvantaged, sexual and gender minority, and physically disabled) (16).

A single reviewer (SV) conducted an initial screening of all data to identify any discrepancies in the results. These disagreements were resolved by consensus among the reviewers. The review was conducted on data that are publicly available to the research community in the NIH RePORTER; specifically, we looked at the research application title, abstract, and the public health relevance statement of the grant application.

## Results

A total of 71 unique titles and abstracts were retrieved via a QVR search. Titles and abstracts of these awards were screened, of which 38 were excluded based on the above criteria. A total of 33 competing awards were included (Appendix B, summary of included awards).

### Grant types

**Figure 1:**
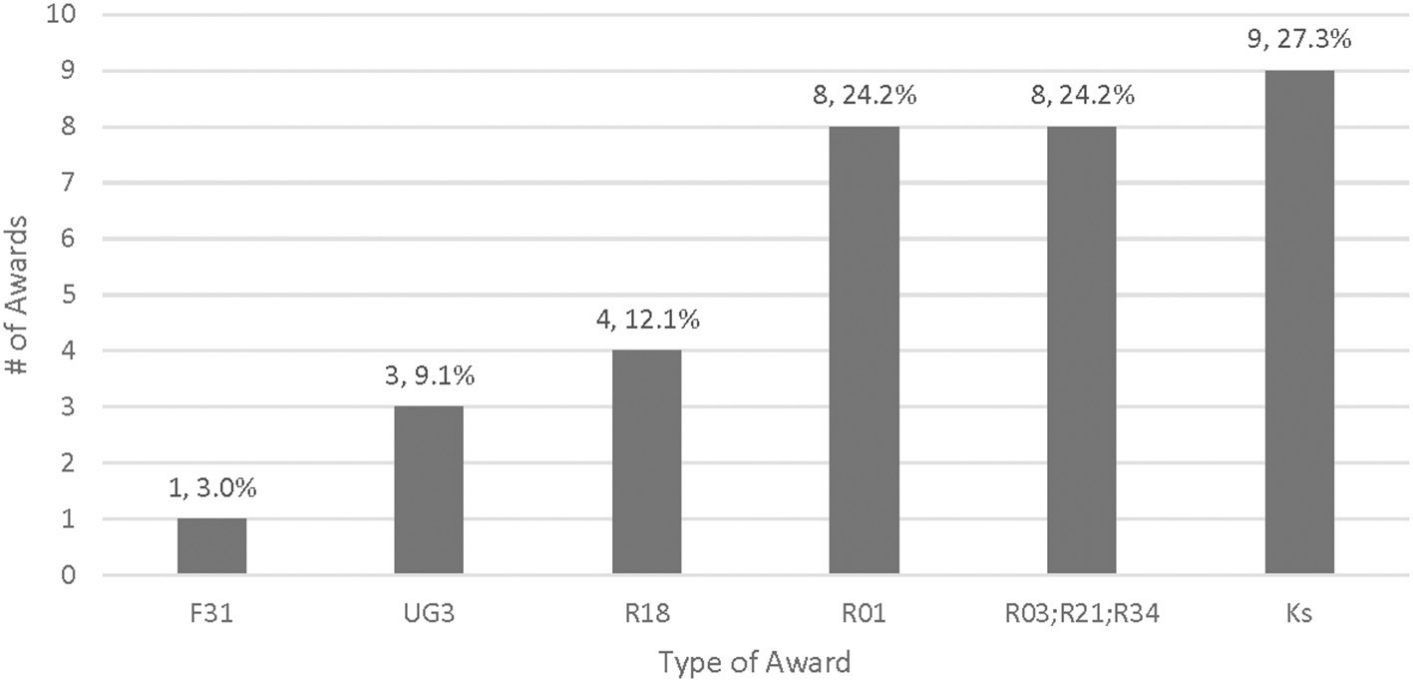
Awards by grant type, 2011–2022 (*n* = 33).

**Figure 2:**
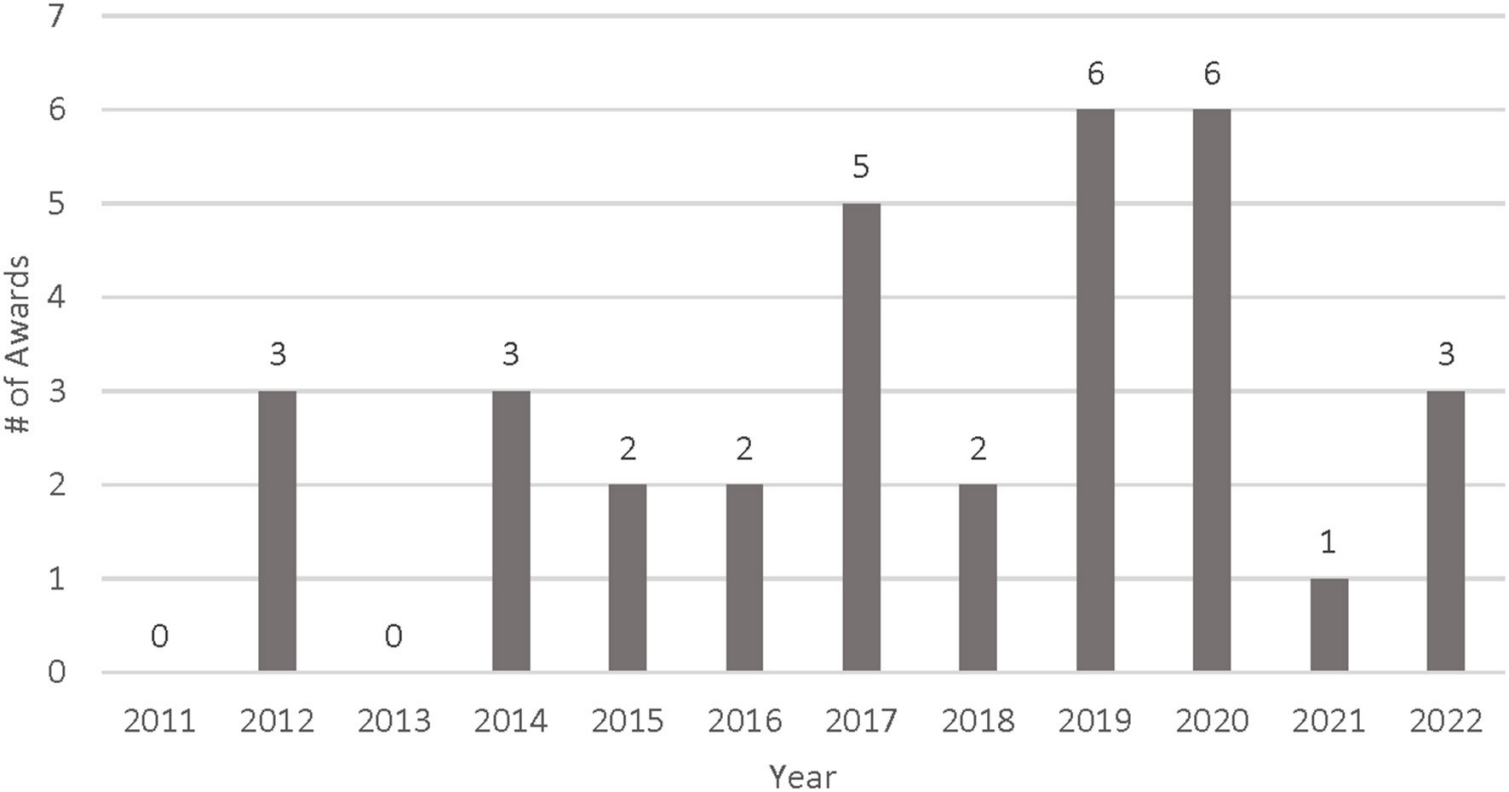
Awards by year initiated, 2011–2022 (*n* = 33).

Almost half of the awards (16/33, 48.5%) were investigator-initiated research, which included R01 Research Project Grants (*n* = 8, 24.2%), R03 Small Grants (*n* = 3, 9.1%), R21 Exploratory/Developmental Research Grants (*n* = 4, 12.1%), and R34 Clinical Trial Planning Grants focused on exploratory and project planning (*n* = 1, 3.0%) (Figure 1). More than a quarter of the awards (*n* = 9, 27.3%) supported research career programs through K funding mechanisms. Four awards were Research Dissemination and Implementation R18 grants (*n* = 4, 12.1%), three were cooperative agreements (U awards) that were awarded in response to a funding solicitation (*n* = 3, 9.1%), and one was an Individual Fellowship for PhD Students (F31, *n* = 1, 3.0%). The leading ICOs for nutrition and dietary IS research from FY 2011 to FY 2022 were the National Heart, Lung, and Blood Institute (NHLBI, *n* = 8, 24.2%) and the National Institute of Diabetes and Digestive and Kidney Diseases (NIDDK, *n* = 8, 24.2%), followed by the National Cancer Institute (NCI, *n* = 7, 21.2%), the National Institute of Minority and Health Disparities (NIMHD, *n* = 4, 12.1%), the *Eunice Kennedy Shriver* National Institute of Child Health and Human Development (NICHD, *n* = 3, 9.1%), the National Institute of Mental Health (NIMH, *n* = 2, 6.1%), and the Fogarty International Center (FIC, *n* = 1, 3.0%). Figure 2 describes the distribution of funded awards by year. Although the inclusion criteria included awards funded in 2011, the search spanned awards from 2012 to 2022. Nearly 70% of the awards were funded between 2017 and 2022, with the last 3 years (2020–2022) accounting for more than a quarter of the grants awarded (*n* = 10, 30.3%).

While most awards were investigator-initiated research applications, four awards responded to a Request for Applications (RFAs), which represent a special interest by one or more ICOs. The first RFA is a funding opportunity for “Late-Stage Implementation Research Addressing Hypertension in Low- and Middle-Income Countries: Scaling Up Proven-Effective Interventions”, which uses a UG3/UH3 two-phase mechanism by NHLBI to facilitate implementation research on hypertension in low- and middle-income countries (17). To date, this RFA has funded three projects, two of which are nutrition and IS awards. The second RFA calls for pilot effectiveness trials for treatment, preventive, and service interventions by NIMH, which involves a clinical trial (18). In response to this funding opportunity, the award, “Adaptation of an Evidence-based Interactive Obesity Treatment Approach (iOTA) for Obesity Prevention in Early Serious Mental Illness” (19), proposes to use combined methods to adapt and pilot test an interactive obesity treatment approach for obesity prevention in early serious mental illness using a design-for-dissemination approach and including a randomized pilot and feasibility clinical trial. The third RFA is an NHLBI K01 mechanism for mentored career development awards to promote faculty diversity in biomedical research (20).

### Research study sample characteristics

Almost half of the funded research (*n* = 16, 48.5%) took place in the United States, while 15.2% was conducted in low- and middle-income countries (LMICs, *n* = 5) in Africa, Latin America, and Asia (Table 1). More than a third of the awards (*n* = 12, 36.4%) did not specify their location in the abstract. Interventions addressed the individual (*n* = 5, 15.2%), organizational (*n* = 9, 27.3%), and community (*n* = 4, 12.1%) levels of the socio-ecological model, with most addressing multiple levels (*n* = 11, 33.3%). Among the grant applications in this analysis, 45.5% focused on study populations comprising adults aged 19–64 years (*n* = 15), while young and elementary school-aged children (2–11 years) accounted for 18.2% (*n* = 6). Infants (0–2 years), adolescents (12–18 years), and older adults (65+ years) were included in research projects in conjunction with other age groups. More than half of the awards (*n* = 18, 54.5%) addressed or specified a focus on health disparities, health equity, or minority health. Predominantly, study designs were experimental (*n* = 12, 36.4%), which included randomized control trials (RCTs), pragmatic RCTs, dynamic wait-listed control designs, and cluster RCTs. Quasi-experimental designs (*n* = 4, 12.1%) included any manipulation without randomization like interrupted time series, and mixed methods (*n* = 4, 12.1%) were also used. Almost a quarter of the awards (*n* = 7, 21.2%) employed more than one study design, including one award focused on measurement development and validation. Eighteen awards used effectiveness-implementation hybrid designs: Type 1 designs (*n* = 7) that tested intervention effects on relevant nutrition or clinical outcomes and collected data on the implementation process; Type 2 designs (*n* = 7) that tested the effects of interventions or implementation strategies with emphasis on both nutrition/clinical and implementation outcomes; and Type 3 designs (*n* = 4) that tested an implementation strategy while observing intervention effectiveness (21).

**Table 1:** Characteristics of NIH-funded Nutrition and Implementation Science Awards, 2011–2022 (*n* = 33).

**Categories**	***n***	**%**
**Countries**		
US	16	48.5%
Nigeria	2	6.1%
Dominican Republic	1	3.0%
Peru	1	3.0%
Malawi, Uganda, Kenya, Burkina Faso, Bangladesh and Pakistan	1	3.0%
Not stated in the abstract	12	36.4%
**Age**		
Young and elementary school children (2–11 years)	6	18.2%
Adults (19–64 years)	15	45.5%
Across ages^*^	3	9.1%
Not stated in the abstract	9	27.3%
**Study design**		
Experimental	12	36.4%
Quasi-experimental	4	12.1%
Mixed methods	4	12.1%
Observational	3	9.1%
Pre-post	1	3.0%
More than one design^**#**^	7	21.2%
Not stated in the abstract	2	6.1%
**Study setting**		
Healthcare	7	21.2%
Childcare	5	15.2%
Community	4	12.1%
Digital	3	9.1%
Faith-based	2	6.1%
Retail	2	6.1%
Workplace	1	3.0%
School	1	3.0%
More than one	3	9.1%
Not stated in the abstract	5	15.2%

^*^One each: children (0–11 years); children and adolescents (0–18 years); adults (19+ years).

^**#**^Experimental and mixed methods (*n* = 2); quasi-experimental and mixed methods (*n* = 1); mix of quasi-experimental, observational, and mixed methods (*n* = 1); systems science modeling and comparative risk assessment model (*n* = 1); markov model with monte carlo simulation (diet cancer outcome model) (*n* = 1); experimental; mixed methods; multiphase optimization strategy (*n* = 1).

### Diet and nutrition intervention research characteristics

A wide range of evidence-based interventions included both face-to-face and digital components. Some interventions included the implementation of established guidelines, such as the WHO Best Buy SHAKE package for salt reduction (UG3HL152381) (22). Others developed novel interventions, one of which is exploring eating behaviors through pilot studies to develop interventions for improving the nutritional environment (K01HL147882). More recently, awards have taken a policy-focused approach by evaluating variations in the implementation of federal nutrition policies during the COVID-19 pandemic (CA260023) or developing and testing an implementation strategy to support the adoption of stronger nutrition standards outlined in the federal Child and Adult Care Food Program (DK125278). Culturally tailored interventions engaged community partners to develop and adapt their interventions. Studies most often took place in healthcare settings (*n* = 7, 21.2%), childcare settings (*n* = 5, 15.2%), and community settings (*n* = 4, 12.1%).

Evidence-based activities related to nutrition and obesity were often part of larger, multicomponent interventions. More than half of the awards employed dietary and lifestyle interventions using tailored self-management approaches (*n* = 17, 51.5%), whereas 24.2% of the awards focused on diet quality and healthy eating patterns (*n* = 8). Few awards explored food insecurity (*n* = 2, 6.1%), symptomatic management of cancer among survivors (*n* = 2, 6.1%), and malnutrition (*n* = 1, 3.0%). While nearly half of the awards focused on obesity (*n* = 15, 45.5%), 15.2% of funded awards explored nutrition-related cardiovascular diseases (*n* = 5) and 9.1% of awards focused on diabetes (*n* = 3). Some of the awards explored more than one nutrition topic. For instance, one award looked at a lifestyle intervention to improve obesity and diabetes risk for immigrant workers at agricultural worksites (R18DK096429).

### Implementation outcomes

Among the awards, most measured more than one IS outcome (*n* = 21, 63.6%). Of the outcomes assessed, feasibility (*n* = 16, 20.3%) was the most defined and measured implementation outcome, followed by costs (*n* = 13, 16.5%) and adoption (*n* = 12, 15.2%) (Table 2). Scale-up was not examined in any of the awards. It is worth noting that three of the four Research Dissemination and Implementation grants (R18) exclusively measured costs, while the cooperative agreements included, on average, five separate implementation outcomes related to nutrition.

**Table 2:** Implementation characteristics of NIH-funded Nutrition and Implementation Science awards (*n* = 33), 2011–2022.

	***n***	**%**
**Implementation outcomes (*****n*** = **79)**		
***Coding individual outcomes across all awards***.		
Feasibility	16	20.3%
Costs	13	16.5%
Adoption	12	15.2%
Acceptability	11	13.9%
Sustainability	10	12.7%
Fidelity	9	11.4%
Adaptation	3	3.8%
Reach	2	2.5%
Penetration	1	1.3%
Appropriateness	1	1.3%
Scale-up	0	0.0%
Not stated in the abstract^*^	1	1.3%
**Implementation focus area (*****n*** = **33)**		
Pre-implementation	7	21.2%
Implementation process description	8	24.2%
Implementation strategy/strategies testing	19	57.6%
Dissemination strategy testing	0	0%
Sustainability	3	9.1%
De-implementation	1	3.0%
IS measurement development	1	3.0%
Scale-up	0	0%
**Implementation science theory, model, and frameworks (*****n*** = **22)**		
***Coding each use of a theory, model, or framework across all awards***.		
Reach, effectiveness, adoption, implementation, maintenance (RE-AIM)	8	36.4%
Consolidated framework for implementation research (CFIR)	3	13.6%
Exploration, preparation, implementation, sustainment (EPIS)	2	9.1%
Interactive systems framework	2	9.1%
Practical, robust implementation and sustainability model (PRISM)	1	4.5%
Quality implementation framework	1	4.5%
Behavioral change wheel	1	4.5%
Dynamic adaptation process framework	1	4.5%
Intervention mapping	1	4.5%
Theoretical domains framework	1	4.5%
Dynamic sustainability framework	1	4.5%
**Study settings (*****n*** = **33)**		
Healthcare	7	21.2%
Childcare	5	15.2%
Community	4	12.1%
Digital	3	9.1%
Faith-based	2	6.1%
Retail	2	6.1%
Workplace	1	3.0%
School	1	3.0%
More than one setting	3	9.1%
Not stated in the abstract	5	15.2%

^*^One study focused on an implementation determinant (organizational readiness) and was included due to use of an implementation science framework.

This review identified six unique implementation focus areas among all 33 awards: pre-implementation, implementation process description, implementation strategy testing, de-implementation, sustainability, and IS measurement development [(15), Table 2]. Five awards (15.2%) addressed more than one focus area relating to different parts of their research projects. More than half of all awards were for testing implementation strategies (*n* = 19, 57.6%). Seven awards (21.2%) focused on the pre-implementation phase that addressed the efficacy, effectiveness, and/or cost-effectiveness of an intervention or adapting an intervention (23). Awards describing the implementation process (including identifying barriers and facilitators) and considering aspects of intervention sustainability comprised 24.2% (*n* = 8) and 9.1% (*n* = 3) of the awards, respectively. Only one award was for developing an IS measurement (3.0%), and only one addressed de-implementation (3.0%).

Among the 16 awards that referenced an established IS theory, model, or framework to guide their work, the Reach, Effectiveness, Adoption, Implementation, Maintenance (RE-AIM) framework, either alone or in conjunction with another framework, was the most cited, accounting for over a third of the referenced frameworks (*n* = 8, 36.4%). The Consolidated Framework for Implementation Research (CFIR, *n* = 3, 13.6%), the Exploration, Preparation, Implementation, and Sustainment Framework (EPIS, *n* = 2, 9.1%), and the Interactive Systems Framework (*n* = 2, 9.1%) were also referenced by multiple awards. The remaining seven frameworks were utilized only once. Seventeen of the 33 award abstracts (51.5%) did not report an IS framework, model, or theory in the abstract.

## Discussion

This analysis of the NIH diet and nutrition research and IS grants portfolio funded between fiscal years 2011 and 2022 found that, while research grants made up the majority of awards when broken down by grant mechanism, there was a fairly even distribution of career development grants, R03s, R21s, and R34s that are more exploratory grant mechanisms of smaller budgets and shorter timeframes and 5-year R01s that are larger in scope and scale and testing a trial or hypothesis. This range of grant mechanisms indicates an opportunity for growth. The number of awards funded through implementation research funding announcements, specifically the PAR “Dissemination and Implementation Research in Health” funding opportunities, was much lower than the other grant mechanisms in this portfolio analysis (24, 25). Funding for IS and nutrition research spiked in 2019 and 2020. Despite a general increase in funding since 2017, only two grants were awarded in FY 2018 and only one in FY 2021. The overall increase in funding to support IS and nutrition in 2017, 2019, and 2020 is encouraging, though the slow growth in 2021 and 2022 is concerning.

While the US hosted all the award types previously mentioned, the five awards that conducted studies in LMICs, which made up more than 15% of the nutrition and IS nutrition portfolio, were limited to career support awards (Ks, *n* = 2), a short-term research grant (R03, *n* = 1), and cooperative agreements (UG3, *n* = 2). None of these were the larger, long-term research projects, specifically research dissemination and implementation grants. In comparison, direct foreign awards only accounted for ~1% of NIH's overall grant portfolio in 2020. Unlike the specific foreign awards identified in this analysis, direct foreign awards across NIH tend to vastly favor long-term research, with less than a quarter of the awards for career development grants being much less likely to support short-term exploratory awards. This contrast between the short-term research and career development awards found in this analysis and the long-term research funding to direct foreign awards across the NIH highlights an area of growth for IS global nutrition.

Most of the grants studied adult populations, while fewer awards focused on children. This distribution likely reflects the scope of these research projects focused on nutrition and diet as part of chronic disease prevention and treatment.

Although specific dietary or nutritional behaviors were difficult to identify from publicly available abstracts, we observed that studies frequently examined obesity and nutrition in tandem. Notably, 18 (54.5%) of the awards were coded as addressing health disparities or health equity. These grants focused on outcomes like feasibility, adoption, and adaptation and most often included a culturally tailored nutrition or dietary intervention. Many of these awards focused on tailoring or targeting an evidence-based intervention for an underserved population, highlighting the researchers' importance in addressing health disparities, health equity, and minority health (26, 27). This finding aligns with the IS field's growing emphasis on addressing health equity and social determinants of health through the use of equity-centered IS frameworks, approaches (including community-engaged research), and measures (26–29).

The majority of awards assessed feasibility, acceptability, cost, and adoption and few (a little more than 10%) assessed sustainability, but none addressed scale-up. Awards included in the portfolio in early-stage implementation more frequently included the following measures: feasibility and acceptability, which focus on ensuring that an intervention is suitable to the population of interest; cost, including assessments of cost-effectiveness and cost-benefits; and adoption, which measures the uptake of an intervention (14). In contrast, sustainability and scale-up are generally addressed later in the research translation or implementation process once there has been uptake and demonstrated effectiveness of an intervention. It is worth noting that “scale-up” is also a relatively new outcome not originally included in Proctor's Implementation Outcomes Framework (14), which may explain its absence from some of the older awards.

Less than half of the awards included an established IS framework to steer the work with the variability of determinants, evaluation, and other kinds of existing frameworks. RE-AIM, CFIR, and EPIS accounted for most of the referenced frameworks. IS frameworks are important tools for guiding research that is “intended to enhance the generalizability of findings by establishing common concepts and terminologies that can be applied across disparate research studies and settings” (30). The lack of frameworks in the majority of awards may indicate the nascent application of IS in nutrition research. In addition, there may be a need to develop and adapt theories, models, and frameworks for application in diverse settings, including in LMICs and among minority populations (31).

The emergence in the last few years of the NIH Implementation of Nutrition-related Programs, Practices, and Behaviors working group, the USG Global Nutrition Coordination Plan's global nutrition and IS technical working group (32), and the National Collaborative on Childhood Obesity Research IS interest group (33) along with three nutrition workshops held across multiple ICOs at the NIH that included a sustainable IS component (34–36) demonstrates the increasing interest in applying IS to nutrition across the federal funding agencies.

As nutrition and IS research become more transdisciplinary, we anticipate an increase in the number of relevant NIH-funded awards. The recent NIH commentary (10) identifies three scientific opportunities to stimulate IS in nutrition research in alignment with the 2020–2030 Strategic Plan for NIH Nutrition Research, calling for including implementation and dissemination early in the intervention design, developing and testing strategies for equitable implementation of nutrition and diet evidence-based, and building and strengthening capacity and expertise needed to increase the use of IS in nutrition research.

A few limitations are worth noting. First, the analysis was limited to publicly available abstracts. Owing to the limitation of abstracts, certain project details, such as the research location, targeted nutritional behaviors, and the priority population's racial and ethnic makeup, were not coded. Additional details could have been available in the full application (e.g., use of IS frameworks). Second, we limited IS outcomes to those that were specifically labeled, which may have eliminated potentially relevant awards that did not list the outcomes in the abstract.

## Conclusion

The Strategic Plan for NIH Nutrition Research 2020–2030 highlights growing interest and opportunities in nutrition and IS research. Findings from this analysis describe the current funded portfolio of research on nutrition research and IS across the NIH. While the overall number of awards was low, there was a gradual increase in those focusing on evidence-based IS research in nutrition and obesity over time. Notably, several of these awards addressed health disparities. Potential opportunities for growth in the nutrition and IS research portfolio include the following areas: (1) greater use of established IS frameworks, (2) an increase in awards that examine scale-up as an implementation outcome, (3) research focused on adaptation, reach, and contextual influences on implementation across diverse populations to promote equitable implementation, and (4) building capacity to increase the use of IS in clinical and community nutrition research. Enhancing IS knowledge, practice, and scale-up can improve the translation of evidence-based nutrition/dietary interventions into effective practice and address health disparities specifically related to nutrition and diet.

## Author contributions

All authors contributed to the development, data coding and analysis, and drafting of the manuscript and approved the submitted version.
