# Supplementary material for: NIH-supported implementation science and nutrition research: a portfolio review of the past decade
Source: Front Public Health. 2023 Oct 17;11:1235164. doi: 10.3389/fpubh.2023.1235164 (PMC10616832; doi:10.3389/fpubh.2023.1235164)
Supplement: Supplementary file 1 [file Data_Sheet_1.docx]

**Nutrition and Implementation Science Portfolio Analysis**

Code Book

**Primary question**: What are the trends in Implementation Science and Nutrition related funded NIH portfolio?

**Secondary questions**:

- What is the volume of NIH funded nutrition and IS research?
  - What kinds of studies:
    - animal subject code (not sure if there are any); human subject code, clinical trial code?
    - Type of research: basic/translational (not sure if there are any); observational or clinical trials/interventions
  - What NIH funding mechanisms were used? What is the trend by FY?
    - RFA vs investigator-initiated
  - NIH ICOs are funding nutrition and implementation science?
  - NIH ICOs in percent of implementation funding to total nutrition funding (Ratio of IS awards in nutrition awards)
  - Number of type 1 and 2 awards and total awards by FY
  - Total cost by FY (include all types- 1, 2, 3, 5, etc.)
- What implementation strategies were used?
  - What implementation outcomes were assessed (e.g., acceptability, adoption, affordability, appropriateness)?
  - What phase of implementation do the studies address?
  - What IS frameworks, theories, and/or models were used in the studies?
- What areas of nutrition were addressed?
  - What major disease conditions were targeted?
  - What type of interventions were tested?
  - What types of nutrition/diet related behaviors were targeted (if a behavioral intervention)?
- Where did the study interventions take place (e.g., school, community clinic)?
  - How was the intervention delivered (e.g., tech, face-to-face, social media)?
  - What were the study populations (e.g., age group, minority populations)?
  - Where are the studies being funded (e.g., country (global), state, rural/urban)?
- What study designs were used (e.g., IS type, hybrid, experimental, observational)?

**Coding Questions**

1. Rater Name
2. Review #
3. Project #
4. Contact PI Name
5. Should this project be included? (can put comments in “other”)
6. Age range of the population: (select all that apply)
   1. Prenatal
   2. Infants (0-2)
   3. Youth children (2-5)
   4. Elementary age children (5-11)
   5. Adolescents (12-18)
   6. Adults (19-64)
   7. Older adults (65+)
   8. Not stated in the abstract
   9. Other
7. Race of population in the trial/study? (select all that apply)
   1. American Indian or Alaska Native
   2. Asian
   3. Black or African American
   4. Native Hawaiian or Other Pacific Islander
   5. White
   6. Not stated in the abstract
8. Ethnicity of population in the trial/study? (select all that apply)
   1. Hispanic or Latino
   2. Not Hispanic or Latino
   3. Not stated in the abstract
9. Any special populations (e.g., pregnant women, healthcare workers, etc.)
10. Location where the study took place (city, state, country)
11. Type of community (select all that apply)
    1. Urban
    2. Rural
    3. Suburban
    4. Not stated in the abstract
12. What was the evidence-based intervention? (e.g., clinical guideline, policy intervention, behavioral intervention, primary prevention)
13. What was the intervention setting?
    1. Faith-based
    2. Community-based organization
    3. Healthcare setting
    4. Recreation area
    5. School
    6. Home
    7. Health department
    8. Workplace setting
    9. Child welfare setting
    10. Justice settings
    11. Online settings
    12. Other, please specify
    13. Not stated in the abstract
    14. Not Applicable
14. Types of nutrition/diet related behaviors targeted in the intervention? (note: be inclusive and generous in answering this question)
15. What behavior theories was employed? (e.g., socio-ecological model, social cognitive theory)
16. How is the intervention being delivered? Select all that apply.
    1. Social marketing
    2. Mobile technology/Apps
    3. Online
    4. Face-to-face
    5. Other- include in the notes
    6. Not stated in the abstract
    7. Not applicable (no intervention is being delivered)
    8. Other, please specify: (free text)
17. What implementation outcomes are assessed? Outcomes developed based on [Proctor, 2011](https://www.ncbi.nlm.nih.gov/pmc/articles/PMC3068522/); [Proctor, 2009](https://www.ncbi.nlm.nih.gov/pmc/articles/PMC3808121/); and [Reilly, 2020](https://www.ncbi.nlm.nih.gov/pmc/articles/PMC7492593/). Select all that apply.

Note: The outcome must be explicitly state with a clear intent to measure it.

- 1. Acceptability
  2. Adoption
  3. Appropriateness
  4. Costs
  5. Feasibility
  6. Fidelity
  7. Penetration
  8. Sustainability
  9. Scale-up
  10. Not stated in the abstract
  11. Other, please specify: (free text)

1. What implementation strategy or strategies is/are being used, if any? List of strategies are based on [Waltz, 2015](https://www.ncbi.nlm.nih.gov/pmc/articles/PMC4527340/). Select all that apply.
   1. Engage consumers
   2. Use evaluative and iterative strategies
   3. Change infrastructure
   4. Adapt and tailor to the context
   5. Develop stakeholder interrelationships
   6. Utilize financial strategies
   7. Support clinicians
   8. Provide interactive assistance
   9. Train and educate stakeholders
   10. Not stated in the abstract
   11. None
2. What phase(s) of implementation research does the study address? Based on [Neta, 2021](https://aacrjournals.org/cebp/article/30/2/260/72355/Dissemination-and-Implementation-Research-at-the). Select all that apply.
   1. Pre-implementation (e.g., efficacy, effectiveness and/or cost-effectiveness of an intervention; adapting an intervention)
   2. Implementation process description (e.g., barriers/facilitators but excluding hybrid designs)
   3. Dissemination strategy testing
   4. Implementation strategy/strategies testing
   5. Sustainability (e.g., describe sustainability; interventions to support/enhance sustainability)
   6. De-implementation
   7. Implementation science measurement development
   8. Scale-up
   9. Not implementation
   10. Other, please specify: (free text)
   11. Not stated in the abstract
3. What implementation model, theory or framework is being employed in the study? Select all that apply.
   1. None
   2. Consolidated Framework for Implementation Research
   3. RE-AIM (Reach, Effectiveness-Adoption, Implementation, Maintenance)
   4. Diffusion of Innovations
   5. EPIS (Exploration, Preparation, Implementation, Sustainment)
   6. Interactive Systems Framework
   7. PRECEDE/PROCEED
   8. Theory of Organizational Readiness for Change (Weiner’s Model)
   9. Other, please specify: (free text)
4. What type of study design is employed? Select all that apply.
   1. Experimental [Manipulation with randomization (e.g., RCT, pragmatic RCT, dynamic wait-listed control, cluster RCT)]
   2. Quasi-experimental [Manipulation but no randomization (e.g., interrupted time series, non-equivalent control group)]
   3. Observational (e.g., cross-sectional, descriptive)
   4. Pre-post design
   5. Systems science modeling (e.g., Agent Based Modeling, System Dynamics Modeling)
   6. Case Study
   7. Social network analysis
   8. Qualitative methods
   9. Mixed methods
   10. Not stated in the abstract.
   11. Other, please specify: (free text)
5. Are health disparities addressed? Yes/no
6. Which NIH Disparity Population is the focus of the research?
   1. Racial/Ethnic Minorities
   2. Underserved rural
   3. Age-defined
   4. Socioeconomically disadvantaged
   5. Sexual and gender minorities
   6. Physically disabled
   7. Other
   8. Not applicable
7. What is the disparities research focus? (Minority health- within group studies; health disparities- between group studies; health equity- intervention on a social determinant of health and/or take an equity approach and/or include a health equity outcome)
   1. Minority health
   2. Health disparities research
   3. Health equity
   4. Not applicable
8. What is the target of the evidence-based practice, program, or intervention (EBP)?
   1. Primary prevention
   2. Secondary prevention/Screening/Testing
   3. Treatment
   4. Survivorship/Quality of life
   5. Surveillance/Epidemiology
   6. Other, please specify: (free text)
9. At what level is implementation taking place?
   1. Community level
   2. Organizational level
   3. Provider level
   4. Individual/family level
   5. Policy level
   6. Multi-level
   7. Not applicable
