# Supplementary material for: NIH-supported implementation science and nutrition research: a portfolio review of the past decade
Source: Front Public Health. 2023 Oct 17;11:1235164. doi: 10.3389/fpubh.2023.1235164 (PMC10616832; doi:10.3389/fpubh.2023.1235164)
Supplement: Supplementary file 2 [file Data_Sheet_2.docx]

Appendix B. Summary of included nutrition and implementation science awards, 2011–2022 (n = 33)

| Project Number | Contact PI Name | Title | Award Type | Primary NIH IC | Year, Initiated |
| --- | --- | --- | --- | --- | --- |
| R18DK079880 | Eaton, Charles B | Tailored Lifestyle Intervention in Obese Adults within Primary Care Practice | R18 | DK | 2012 |
| R18DK096429 | Schenker, Marc B | Translating Obesity and Diabetes Prevention into the Worksite for Immigrant Popul | R18 | DK | 2012 |
| R18HL112720 | Perri, Michael G | Rural Lifestyle Eating and Activity Program (Rural LEAP) | R18 | HL | 2012 |
| K24DK103992 | Bibbins-Domingo, Kirsten | Hypertension Management in Minority and Low-income Populations | K24 | DK | 2014 |
| R01MH102325 | Bartels, Stephen J | RCT of a Learning Collaborative to Implement Health Promotion in Mental Health | R01 | MH | 2014 |
| R18DK102737 | Wexler, Deborah J | REAL HEALTH-Diabetes: REach Ahead for Lifestyle and Health-Diabetes | R18 | DK | 2014 |
| K23DK104090 | Haemer, Matthew A | Tailoring of Childhood Obesity Screening and Counseling for Latino Families with Preschool-Aged Children | K23 | DK | 2015 |
| R03CA197657 | Perry, Cynthia Kay | Fuerte y Sanas: Adaptation of an Exercise and Nutrition Program for Rural Latinas | R03 | CA | 2015 |
| K23HL133604 | Nicklas, Jacinda Mawson | Iterative development and pilot testing of a mobile health intervention to increase postpartum weight loss in women at increased risk for cardiometabolic disease | K23 | HL | 2016 |
| R01MD011501 | Zhang, Fang Fang | Comparative and Cost-Effectiveness of Population Strategies to Improve Diet and Reduce Cancer | R01 | MD | 2016 |
| K43TW010704 | Odukoya, Oluwakemi | Promoting Physical Activity and Healthy Eating Among Adults in a Faith-Based Setting In Lagos, Nigeria. | K43 | TW | 2017 |
| R01DK115434 | Dabelea, Dana | Reducing risk factors for type 2 diabetes in American Indian youth: Tribal Turning Point | R01 | DK | 2017 |
| R01HD091136 | Bastani, Roshan | Addressing Obesity in Early Care and Education Settings | R01 | HD | 2017 |
| R01HL137929 | Ward, Dianne Stanton | A hybrid effectiveness-implementation trial of Go NAPSACC: a childcare-based obesity prevention program | R01 | HL | 2017 |
| R21CA215668 | Stolley, Melinda R | Avanzando Juntas: Adapting an evidence based weight loss program for Hispanic Breast Cancer Survivors | R21 | CA | 2017 |
| K23HD096204 | Herrick, Cynthia J | Clinic to Community Connections: Enhancing Prenatal to Postpartum Care Transitions to Prevent Type 2 Diabetes in Low Income Women with Gestational Diabetes | K23 | HD | 2018 |
| R03DK117197 | Swindle, Taren | De-Implementation of Detrimental Feeding Practices in Childcare- Resubmission | R03 | DK | 2018 |
| K01HL147882 | Fuster, Melissa | Applying Innovative Approaches to Design and Implement an Intervention to Improve Cardiovascular Health in Hispanic/Latino communities through Restaurants | K01 | HL | 2019 |
| R03HD099270 | Means, Arianna Rubin | Prevalence and correlates of pediatric guideline deviation across 9 health facilities | R03 | HD | 2019 |
| R21CA236057 | Sun, Virginia Chih-Yi | Altering Intake and Managing Symptoms in Rectal Cancer Survivors: A Multimodal Diet Modification Intervention for Bowel Dysfunction | R21 | CA | 2019 |
| R21CA237984 | Swindle, Taren | Sustainability of Nutrition and Physical Activity Interventions in Childcare | R21 | CA | 2019 |
| R34MH118395 | Nicol, Ginger Ellen | Adaptation of an Evidence-based Interactive Obesity Treatment Approach (iOTA) for Obesity Prevention in Early Serious Mental Illness: iOTA-eSMI | R34 | MH | 2019 |
| R01CA228527 | Fernandez, Maria Eulalia | Development and Validation of a Measure of Organizational Readiness (motivation x capacity) for Implementation | R01 | CA | 2019 |
| K23MD015088 | Shah, Megha | Better Together: Leveraging primary care and social network resources to create a patient-centered approach to improve diabetes among immigrant communities | K23 | MD | 2020 |
| K23MD015267 | Byhoff, Elena | Increasing SNAP enrollment in a diverse Latinx community | K23 | MD | 2020 |
| R01HL152714 | Schoenberg, Nancy E | Implementing an evidence-based mHealth diet and activity intervention: Make Better Choices 2 for rural Appalachians | R01 | HL | 2020 |
| UG3HL151309 | He, Jiang | Community Health Worker-Led Church-Based Intervention for Eliminating Cardiovascular Health Disparities in African Americans | UG3 | HL | 2020 |
| UG3HL152371 | Malaga, German | Addressing HyperteNsion and Diabetes through Community-Engaged Systems in Puno, Peru (ANDES study) | UG3 | HL | 2020 |
| UG3HL152381 | Ojji, Dike Bevis | Evaluating the Implementation and Scale-Up of Nigeria National Sodium Reduction Program | UG3 | HL | 2020 |
| K01DK125278 | Kenney, Erica Lauren | Identifying an implementation strategy to maximize the public health nutrition impact of the Child and Adult Care Food Program | K01 | DK | 2021 |
| R21CA260023 | Lane, Hannah G | Expanding the impact of cancer prevention policies through collaborative implementation research: a qualitative secondary analysis of federal child nutrition assistance policies during COVID-19 | R21 | CA | 2022 |
| F31CA268894 | Puklin, Leah | Bridging the Gap: Evaluating why and how to implement lifestyle programs for cancer patients into routine clinical oncology care | F31 | CA | 2022 |
| R01MD016908 | Gany, Francesca M | Taxi ROADmAP (Realizing Optimization Around Diet And Physical activity) | R01 | MD | 2022 |
